# Conducting Research in Palliative Care as Viewed by Interprofessional Care Teams: Insights from a Cross-Sectional Survey

**DOI:** 10.1089/pmr.2024.0099

**Published:** 2025-06-25

**Authors:** Coralie Roux, Sophie Pautex, Federica Bianchi, Lisa Hentsch

**Affiliations:** ^1^ Medical Faculty, Geneva University, Geneva, Switzerland.; ^2^ Division of Palliative Medicine, Department of Rehabilitation and Geriatrics, Geneva University Hospitals, Geneva, Switzerland.

**Keywords:** barriers, facilitators, interprofessional team, palliative care, research

## Abstract

**Introduction:**

Conducting studies in palliative care can be challenging. It has been highlighted that the interprofessional team may have their own reasons for not engaging in research projects. We aimed to identify barriers and facilitators to the involvement of palliative care team members in research projects.

**Method:**

We used a cross-sectional online survey with qualitative and quantitative components to identify barriers and facilitators encountered by palliative care health professionals. Participants were physicians, nurses, nurse assistants, physiotherapists, dieticians, and occupational therapists working in the Division of Palliative Medicine of the Geneva University Hospitals in Switzerland. Data were analyzed using descriptive statistics and content analysis for the open-ended questions.

**Results:**

A total of 107 questionnaires were sent, and 51 participants (48%) provided responses, of whom 75% expressed an interest in research, although only 47% had previously taken part in a research project. The most cited barriers were a lack of training on how to conduct studies, a lack of time, and a lack of funding. The main facilitators were the recognition that research enhances the quality of care and the belief that patients should be respected in their autonomy and given the opportunity to participate in research projects.

**Conclusion:**

The interprofessional palliative care team would benefit from time, funds, and training in order to enhance a research culture within the team. The establishment of an interprofessional network to guide and share experiences would also be a good way to promote this culture.

## Introduction

Research plays a crucial role in advancing the evidence base of palliative care, enhancing the quality of care, and improving the lives of patients and their caregivers, just like in any other medical domain. However, there has been a noticeable lack of research in palliative care compared with other fields.^1,2^ Conducting studies in palliative care has proven particularly challenging, resulting in a predominantly descriptive nature of published research.^3,4^ Randomized trials are scarce, and studies often involve a limited number of patients, complicating the interpretation and inference of findings.^5^ Palliative care professionals often rely on clinical experience and expert opinion rather than concrete scientific evidence.^6–8^ The unique characteristics and complexities associated with palliative care, such as unpredictable illness trajectories and the diverse needs of patients, contribute to the difficulties encountered in conducting rigorous research.^9–12^ Ethical considerations surrounding end-of-life management can also add challenges when designing and conducting research studies.^10,13^

This research gap in palliative care has prompted research teams worldwide to investigate its underlying causes. Several factors have been identified, many associated with the patients and their unique circumstances.^14,15^ Patients’ frailty, their expressed desire for undisturbed end-of-life experiences, and concerns voiced by their families regarding their well-being are some examples. However, studies on this subject have also revealed that, contrary to assumptions, many patients are interested in participating in research projects.^16^ Their motivations may include altruistic reasons, seeking meaning in their end-of-life journey, or hoping to derive personal benefits from these studies.^17,18^

The scientific literature also indicates that palliative care providers often hesitate to involve these vulnerable patients in projects with uncertain outcomes, acting as gatekeepers.^19–21^ Therefore, it seems important to investigate health care professionals’ perspectives in order to overcome barriers to and possibly erroneous perceptions around conducting research in this field.^22^ Among the barriers encountered by health care personnel worldwide are fears of upsetting patients, a lack of training in conducting research, concerns about appearing insensitive to patients’ suffering, assumptions that patients may not want to participate, and challenges related to funding, time constraints, and personal motivations, among others.^4,23^

This study aimed to determine the barriers and facilitators to the participation of health care professionals in research projects in the Division of Palliative Medicine of a large university hospital. Our secondary objective was to explore areas of development to improve the integration of a research culture and thus improve the quality of care delivered to patients.

## Methods

### Study design

A cross-sectional web-based survey was conducted and reported according to the Consensus-Based Checklist for Reporting Survey Studies to ensure comprehensive reporting and adherence to rigorous standards. This study was conducted from September 2021 to December 2021.

### Sample characteristics

The survey was conducted in the Division of Palliative Care of the Geneva University Hospitals (HUG), in Geneva, Switzerland, which is the biggest Division of Palliative Care in Switzerland with a total of 36 inpatient beds located in three units on two separate sites. The Division of Palliative Care also includes a specialist hospital-based team, a home-based team, and an outpatient team composed of government-funded professionals specifically trained in palliative care.

The target professionals were physicians, nurses, nurse assistants, physiotherapists, dieticians, social workers, psychologists, and occupational therapists working for the Division of Palliative Medicine of the HUG.

### Survey development

A survey (Appendix 1) was designed following a literature search that helped us recognize previously identified barriers and facilitators.^4,21–24^ The database used was primarily PubMed, and the principal keywords were “palliative care,” “research,” “interprofessional,” “clinical research,” “clinical trial,” “palliative care patients,” “end of life,” “barriers,” “challenges,” “difficulties,” and “facilitators.” A barrier is defined as something that prevents or makes it harder for the health care professional to do or participate in a research project, and a facilitator is something that encourages or enables the health care provider to do research. We identified 18 barriers and 7 facilitators in the literature search. The final questionnaire used in the study, written in French, consisted of 15 questions, some with multiple components, with answers presented as multiple-choice or Likert-scale, designed to capture participants’ perceptions of these barriers and facilitators. The Likert-scale options ranged from “disagree” to “partly disagree,” “neutral,” “partly agree,” and “agree.” In addition to the structured questions, participants could provide open-ended comments or share any additional barriers or facilitators they experienced in their practice. Questions 1–9 aimed to gather socioprofessional context and provide an understanding of participants’ backgrounds and perspectives related to the research. This included their professional roles, years of experience, educational background, and previous research involvement. Questions 10 and 11 focused on specific barriers identified in existing literature on palliative care research. Participants were asked to reflect on these barriers and indicate their agreement or disagreement. Questions 12 and 13 explored the facilitators identified in the literature, and participants were asked to express their agreement or disagreement. Questions 14 and 15 centered on participants’ openness to potential solutions for the future.

The survey was then pretested on a sample of three health care professionals, two physicians and a nurse, working in palliative medicine and therefore representative of the sample population.

### Survey administration

To ensure comprehensive representation of the target population, the questionnaire was distributed via e-mail to all members of the interprofessional team within the Division of Palliative Medicine of the HUG. As specified in the e-mail, clicking through to progress to the survey indicated consent. All participants received a follow-up reminder 1 month later, with a final reminder sent an additional month later. Respondents were explicitly instructed to complete the questionnaire only once. No compensation was offered for survey completion.

Upon submission, responses were automatically captured and securely stored in an Excel database. To uphold confidentiality and privacy, no personal identifiers, such as names or e-mail addresses, were collected or retained.

Prior to the survey’s distribution, a brief informational session was conducted at each of the three palliative care units of the HUG. These sessions introduced the research project, outlined its objectives, and emphasized the importance of gathering input from health care professionals working in palliative care. This approach aimed to enhance participant engagement and underscore the value of their contributions.

### Analysis

To ensure confidentiality, personal identifiers such as e-mail addresses were not collected. The survey data were thoroughly cleaned to ensure data quality and integrity. This process involved checking for missing values and inconsistencies. Invalid responses were excluded from the analysis. Missing data were not imputed. Categories from the Likert-scale responses were collapsed with “disagree” to “partly disagree” presented as “disagree” and “partly agree “and “agree” presented as “agree.” We conducted a descriptive analysis of frequencies, percentages, and means for each question. Open-ended questions, generally used to inform or expand on quantitative data, were analyzed using an inductive thematic analysis.^25^

Data from the open-ended questions were exported into an Excel spreadsheet. After familiarization, a coding framework was conceived by C.R. and L.H., who then attributed different codes and themes to the data using a content analysis based on Braun and Clarke’s framework for thematic analysis.^25^

## Results

### Participants

An e-mail with a link to the web-based survey was sent to 107 health care professionals. Fifty-one (47%) completed the questionnaire. Six participants answered the questionnaire only partially. Their results were included in the analysis. Open-ended questions were often answered with only one word as a way to expand or justify answers provided in the survey.

#### Characteristics of health care professionals

**FIG. 1.**
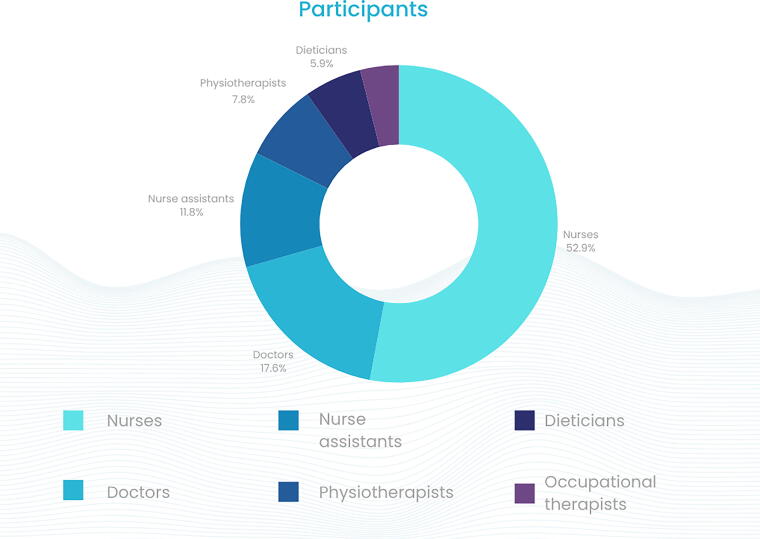
Participants’ professional role in the palliative medicine division.

A significant majority of respondents were women, representing 82% of total participants. Twenty-seven nurses, nine doctors, six nurse assistants, four physiotherapists, three dieticians, and two occupational therapists participated in the study. Notably, no social workers or psychologists answered the questionnaire. Participants demonstrated a breadth of professional experience in the medical field, ranging from 2 to 37 years. Similarly, their involvement in palliative care varied, with durations ranging from 3 weeks to 27 years (Fig. 1).

#### Research interest and experience in research

Among the participants, a significant proportion (74%) expressed interest in engaging in research activities. However, the data revealed that only 47% of the respondents had prior experience participating in research projects. For those involved, their level of engagement varied, with one-third of participants (30%) having contributed to one to three projects. Only one respondent had contributed to over 10 projects, showcasing that person’s active research experience. Regarding awareness of research projects within their units, the findings indicate that half of the participants (50%) were aware of ongoing research initiatives. In the open-ended questions, most participants held a positive view of research, recognizing its usefulness and impact on the quality of care.

### Barriers identified by participants

Most participants expressed concerns about several key aspects, including a lack of training in conducting studies (75%), inadequate funding for projects (55%), time limitations due to heavy workloads (79%), and the complexity of conducting long-term studies in the context of rapidly deteriorating patient conditions. Additionally, results of open-ended questions highlighted participants’ need for improved recognition and support for nurses’ involvement in research, perceiving research as being conducted by physicians only. One-third (33%) of health professionals agreed when asked if they thought the patient’s family would be reluctant to involve their loved ones in research projects. Also, one-third (33%) agreed that study procedures, such as additional blood tests or questionnaires, would be too invasive for patients. Some also mentioned not being approached to participate in studies and a lack of awareness about existing research projects. The survey responses revealed a high degree of nuance, with some viewing these factors as barriers, while others reported no significant difficulties. This variability underscores the complexity of these potential barriers to research participation.

More than one-third of participants (35%) agreed with the fact that palliative care patients’ frailty is a barrier to their participation. Regarding sensitivity to patient suffering, a significant majority (81%) expressed a lack of fear in appearing insensitive. Ethical concerns regarding research on palliative care patients were minimal, as most of the participants (87%) did not express fear of moral implications. In addition, the majority of participants (73%) were not concerned that patients would participate solely due to expectations of financial compensation. Barriers are presented in Table 1.

**Table 1.** Research Barriers Expressed by Health Care Professionals Working in Palliative Care, *n* (%)

	Agree	Partly agree	Neutral	Partly disagree	Disagree
You feel that palliative care patients are too fragile to participate.	1 (2.04%)	5 (10.20%)	11 (22.45%)	15 (30.61%)	17 (34.69%)
You feel that the patients need to be left alone.	1 (2.04%)	9 (18.37%)	10 (20.41%)	17 (34.69%)	12 (24.49%)
You think the family will be reluctant.	1 (2.08%)	15 (31.25%)	13 (27.08%)	11 (22.92%)	8 (16.67%)
You lack training in how to conduct a study.	23 (46.94%)	14 (28.57%)	3 (6.12%)	3 (6.12%)	6 (12.24%)
You are afraid of appearing insensitive to the patient’s suffering.	0	6 (12.24%)	3 (6.12%)	9 (18.37%)	31 (63.27%)
You think the patient will not want to participate.	2 (4.08%)	9 (18.37%)	13 (26.53%)	17 (34.69%)	8 (16.33%)
You feel that there is a lack of funding for projects.	17 (34.69%)	10 (20.41%)	18 (36.73%)	3 (6.12%)	1 (2.04%)
You don’t have enough time to do studies in addition to your current workload.	32 (65.31%)	11 (22.45%)	2 (4.08%)	1 (2.04%)	3 (6.12%)
You don’t feel like doing research.	4 (8.16%)	2 (4.08%)	15 (30.61%)	8 (16.33%)	20 (40.82%)
You are not offered to participate in studies.	13 (26.53%)	8 (16.33%)	13 (26.53%)	6 (12.24%)	9 (18.37%)
You are not aware of the different existing projects.	14 (28.57%)	11 (22.45%)	7 (14.29%)	10 (20.41%)	7 (14.29%)
You find it unethical to do research on palliative care patients.	1 (2.04%)	0	5 (10.20%)	6 (12.24%)	37 (75.51%)
You find it difficult to communicate well with patients to obtain informed consent.	3 (6.25%)	12 (25.00%)	9 (18.75%)	17 (35.42%)	7 (14.58%)
You are afraid that patients will agree to participate because they feel obligated.	2 (4.08%)	2 (4.08%)	9 (18.37%)	10 (20.41%)	26 (53.06%)
You feel that patients’ conditions can sometimes deteriorate too quickly to conduct long studies.	14 (28.57%)	25 (51.02%)	6 (12.24%)	2 (4.08%)	2 (4.08%)
You find the studies too invasive (additional blood tests, questionnaires, etc.).	2 (4.08%)	14 (28.57%)	24 (48.98%)	4 (8.16%)	5 (10.20%)
You feel that you are taking advantage of the patient.	0	4 (8.16%)	4 (8.16%)	7 (14.29%)	34 (69.39%)
You have the impression that the results of the studies are not applied in the clinic anyway.	1 (2.04%)	5 (10.20%)	20 (40.82%)	11 (22.45%)	12 (24.49%)

### Facilitators identified by participants

The majority (75%) of participants indicated that their motivation to participate in research facilitated their involvement. Notably, an overwhelming majority of participants (97%) recognized the value of research as a pathway to enhance the quality of care provided to patients. Health care providers expressed confidence in the benefits of research for patients, with many (86%) believing that patients would derive positive outcomes from their research endeavors. Furthermore, participants emphasized that patients could find meaning in their end-of-life journey by participating in research projects. Regarding patient autonomy, most participants (86%) believed that patients should have the freedom to choose whether or not they wish to participate in various research projects. Interestingly, only a little more than half of the participants (60%) trusted the ethics committee to ensure that only ethically sound research projects were approved. Facilitators are presented in Table 2.

**Table 2.** Research Facilitators Expressed by Health Care Professionals Working in Palliative Care, *n* (%)

	Agree	Partly agree	Neutral	Partly disagree	Disagree
You are motivated to participate in research.	18 (40.00%)	16 (35.56%)	9 (20.00%)	1 (2.22%)	1 (2.22%)
You think patients will benefit from research.	21 (46.67%)	18 (40.00%)	5 (11.11%)	1 (2.22%)	0 (0.00%)
You believe that research can improve the quality of care.	36 (80.00%)	8 (17.78%)	1 (2.22%)	0 (0.00%)	0 (0.00%)
You believe that patients can make sense of their end of life through their participation in research projects.	14 (31.11%)	20 (44.44%)	9 (20.00%)	2 (4.44%)	0 (0.00%)
You believe that patients are autonomous (as long as they have their capacity for discernment) and that it should not be decided for them if they can or cannot participate in various projects.	33 (73.33%)	6 (13.33%)	3 (6.67%)	3 (6.67%)	0 (0.00%)
You have research experience or a network to help you.	7 (15.56%)	10 (22.22%)	8 (17.78%)	3 (6.67%)	17 (37.78%)
You trust ethics committees to only accept correct research projects.	17 (37.78%)	10 (22.22%)	12 (26.67%)	3 (6.67%)	3 (6.67%)

When allowed to comment about encountering other facilitators in their practice, three participants shared more about their perspectives. Two individuals mentioned that being in a hospital setting where research is one of the primary missions and a prominent activity has been helpful to them.

Additionally, one participant highlighted the importance of support from management as a facilitator.

### Factors and improvements that would help participants engage in research

Several common themes emerged from respondents who shared their perspectives in the open-ended questions (*n* = 39). Unsurprisingly, 21 participants expressed needing more time to engage in research activities. Additionally, six participants emphasized the importance of receiving more information and suggested having a research symposium within their department. Furthermore, 12 participants highlighted the importance of research training and wished to benefit from the assistance of more experienced colleagues, suggesting free coaching sessions with regular appointments. Another critical aspect mentioned by 10 participants was the desire for greater opportunities to participate in research, particularly by integrating professions other than physicians. Some even expressed the need for research topics more closely aligned with nursing. They also mentioned that it would be beneficial if their supervisor directly suggested research themes. Regarding resources, three participants called for increased funding, while access to free reference management programs and statistical analysis software were suggested. Finally, two participants emphasized the importance of additional staff covering their responsibilities while they engage in research projects.

Most participants (75%) indicated they would be receptive to suggested solutions within their division.

### Results by professions

All professions were interested in research (with a range going from 100% for physiotherapists to 50% for dieticians and occupational therapists) (Table 3). Physicians were the most likely to have participated in research (88%) and complained the least about the lack of training. Physicians, nurses, physiotherapists, and dieticians were partially aware of research projects in the unit. Occupational therapists, on the contrary, were unaware of the projects being carried out. Finally, the majority (55% to 100%) of the different professional groups showed interest in solutions to implement more research in their units, led by the physiotherapists, who answered 100% positively.

**Table 3.** Research Interest and Awareness, *n* (%)

	Yes	No	I don’t know
Are you interested in research?			
Physicians	7 (77.78%)	2 (22.22%)	0 (0.00%)
Nurse	21 (77.78%)	2 (7.41%)	4 (14.81%)
Nurse assistant	4 (66.67%)	1 (16.67%)	1 (16.67%)
Physiotherapist	4 (100.00%)	0 (0.00%)	0 (0.00%)
Dietician	1 (33.33%)	1 (33.33%)	1 (33.33%)
Occupational therapist	1 (50.00%)	1 (50.00%)	0 (0.00%)
Did you already participate in research projects?			
Physicians	8 (88.89%)	1 (11.11%)	N/A
Nurse	12 (44.44%)	15 (55.56%)	N/A
Nurse assistant	0 (00.00%)	6 (100.00%)	N/A
Physiotherapist	2 (50.00%)	2 (50.00%)	N/A
Dietician	1 (33.33%)	2 (66.67%)	N/A
Occupational therapist	1 (50.00%)	1 (50.00%)	N/A
Did you know about the existence of research projects in your unit?			
Physicians	5 (55.56%)	4 (44.44%)	N/A
Nurse	14 (51.58%)	13 (48.15%)	N/A
Nurse assistant	2 (33.33%)	4 (66.67%)	N/A
Physiotherapist	2 (50.00%)	2 (50.00%)	N/A
Dietician	2 (66.67%)	1 (33.33%)	N/A
Occupational therapist	0 (00.00%)	2 (100.00%)	N/A
If approaches were implemented in your unit to facilitate research, would you be interested?			
Physicians	6 (66.67%)	1 (11.11%)	2 (22.22%)
Nurse	20 (83.33%)	0 (0.00%)	4 (16.67%)
Nurse assistant	2 (50.00%)	1 (25.00%)	1 (25.00%)
Physiotherapist	4 (100.00%)	0 (0.00%)	0 (0.00%)
Dietician	2 (66.67%)	0 (0.00%)	1 (33.33%)
Occupational therapist	1 (50.00%)	0 (0.00%)	1 (50.00%)

N/A, not available.

## Discussion

The first aim of our interprofessional survey was to better identify barriers and facilitators related to research in palliative care settings. Although conducted during the COVID-19 pandemic, the response rate was 48%, identical to other survey-based studies.^26^ We therefore believe that the pandemic did not influence our results.

Generally, participants had a favorable view of research and were open to the possibility of participating and contributing more to research. These results are similar to those found in another recent study conducted on the barriers and facilitators to palliative and end-of-life research.^27^

The lack of research in palliative care has been recognized for some time, and it was believed, initially, to be due to the characteristics of end-of-life patients. However, studies focusing on patients have revealed that they are willing to participate in research.^18^ In studies testing patients’ willingness to participate in research, it was found that patients were more willing than expected, leading to a shift in focus toward health care professionals as a potential barrier to this lack of research. The existing literature on the topic is scarce, one hypothesis is that a form of “gatekeeping” occurs, with health care professionals reluctant to include dying patients.^19,20,28^ In our study, barriers related to ethical reasons or representations of the patient at the end of life as fragile and requiring protection were not predominant, as only 12% of participants agreed that palliative care patients were too fragile to be included in studies. Practical problems, such as lack of time, funding, and training, were the most frequently cited challenges. This has been clearly identified in previous studies but also in other settings and is therefore probably not specific to palliative care.^27,29^

In this study, participants other than physicians felt that they were often omitted, not offered the opportunity, and that the studies were primarily conducted by and for physicians, which excluded a significant portion of the interprofessional team, emphasizing the need for greater inclusion.

The objective of this work was to explore potential solutions and the team’s willingness to change the status quo. A total of 75.5% of the participants indicated they would be interested if solutions to promote research participation were offered by their department. Other than more funds and time dedicated to research within their usual schedule, which may not be achievable in heavy workload settings, there was also a demand for training and assistance from mentors. Setting up a professional network to explain, guide, and share research projects could potentially encourage those who do not feel sufficiently proficient in conducting research independently.^30^ Raising awareness about the benefits of research and the desire of patients to participate could also help to encourage those who are not yet interested. The team also pointed out that they do not know enough about current projects, which prevents them from participating. Better communication about existing or future projects via meetings, e-mail, or a regular newsletter could address this issue. Involving nonphysicians in studies will help gain knowledge and focus interest on a broader range of topics as they may choose to investigate subjects not attended to by physicians.^31^

Following this study, several solutions are currently being implemented in the Division of Palliative Medicine of the HUG to address the lack of ongoing research. For example, a research coordinator has been hired and has been working with the team since January 2022 to promote a proactive research culture. In a recent study on this subject, having dedicated research staff was also identified as a key facilitator in increasing research involvement.^27^ The coordinator assists the team in structuring their work and refers them to individuals who can help with their research projects. Additionally, she organizes monthly research meetings for the palliative care division, where each team member can present their work. These meetings are open to all members of the interprofessional team, with a predominance of nurses, physiotherapists, and doctors promoting their ideas. This change has stimulated collaboration and knowledge acquisition regarding current practices. A poster has also been produced with all the publications from the past year with QR codes leading to each article. Finally, a shared file on the HUG’s server has been created with all the current research projects, links to training/information sessions, and grant proposals. This file is open to all members of the interprofessional team and updated by the research coordinator. The mid- to long-term effect of these strategies on the number and quality of publications will have to be assessed. Our study has limitations, the main one being our relatively small sample size. Moreover, our sample comprises health care professionals who agreed to answer a survey about their interest in research, which might have excluded health care professionals who were not involved or interested in research. Therefore, we cannot exclude a selection bias that may influence results by minimizing barriers. Furthermore, using a survey format restricted response possibilities and may have overlooked some relevant points. A qualitative approach or a mixed-method study, such as interviews, could have allowed for further exploration and a more comprehensive understanding of the subject.^27^ Nevertheless, we repeatedly encouraged participants to provide comments and additional relevant information in open-ended questions, which helped gather Supplementary Data. Finally, this study was conducted in three units of the same Division of Palliative Medicine of a university hospital, so the external validity of the results may not apply to health care settings with different research provisions, for instance, in smaller, more rural structures.

## Conclusion

The importance of developing a research culture to improve patients’ quality of care is evident. This study indicates an interest among professionals working in palliative care to engage in research. Although barriers exist, this positive attitude toward research underscores the potential for growth and improvement in palliative care. Institutional promotion of interprofessional research collaboration, increasing funding opportunities, allocating dedicated research positions, and providing training and mentorship may enhance the capacity of the interprofessional team to engage in meaningful research endeavors.

## Supplementary Material

Supplementary Data
