# Supplementary material for: Conducting Research in Palliative Care as Viewed by Interprofessional Care Teams: Insights from a Cross-Sectional Survey
Source: Palliat Med Rep. 2025 Jun 25;6(1):374–81. doi: 10.1089/pmr.2024.0099 (PMC12410426; doi:10.1089/pmr.2024.0099)
Supplement: Supplementary Data [file pmr.2024.0099_supplementary_data.docx]

**Annexes**

Barrières à la recherche clinique dans les unités de soins palliatifs – Le point de vue des soignants

*Questionnaire pour tous les professionnels travaillant dans le service de soins palliatifs (médecins, infirmièr(e)s, aides-soignant(e)s, assistant(e)s sociales, physiothérapeutes, diététicien(ne)s, psychologues, et ergothérapeutes)*

1.
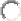
Vous êtes :


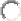

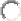
Une femme Un homme Autre

1.
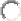

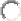
Quelle est votre profession ? Médecin


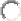

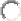
Infirmier.ère Aide -soignant.e


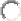

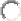

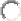

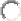

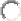
Assistant.e sociale Physiothérapeute Diététicien.ne Psychologue Ergothérapeute Autre

1. Depuis combien de temps exercez-vous dans le domaine médical?


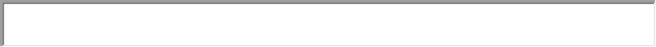


1. Depuis combien de temps travaillez-vous dans les soins palliatifs?


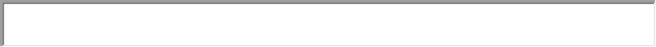


1.
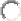

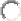
Êtes-vous intéressé.e par la recherche? Oui


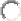
Non

Je ne sais pas

1.
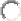

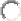
Avez-vous déjà participé à des projets de recherche? oui


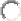
non

Je ne sais pas

Si oui, combien et depuis combien de temps?


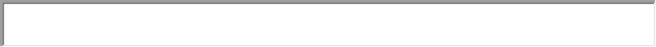


1.
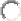
Êtes vous au courant de l'existence de projets de recherche dans votre unité? Oui


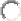
Non

1. Souhaitez vous rajouter des informations sur la recherche à Bellerive et votre activité dans ce domaine?


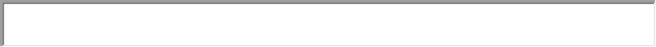


Barrières à la recherche

1. Quel est votre point de vue sur la recherche?


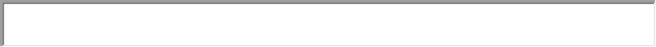


1. Quelles sont les barrières à la recherche?

|  | Je suis en désaccord | Je suis un peu en désaccord | Neutre | Je suis un peu d'accord | Je suis d'accord |
| --- | --- | --- | --- | --- | --- |
| Pour vous, les patients en soins palliatifs sont trop fragiles pour participer | 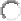 | 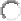 | 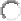 | 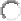 | 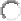 |
| Vous avez l'impression que le patient a besoin de ne pas être dérangé | 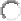 | 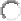 | 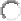 | 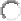 | 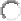 |
| Vous pensez que la famille sera réticente | 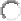 | 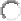 | 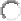 | 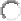 | 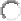 |
| Vous manquez de formation sur la façon de réaliser une étude | 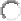 | 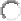 | 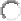 | 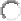 | 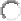 |
| Vous avez peur d'avoir l'air insensible à la souffrance du patient | 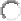 | 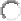 | 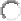 | 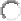 | 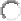 |
| Vous pensez que le patient n'aura pas envie de participer | 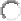 | 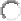 | 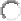 | 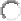 | 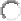 |
| Vous trouvez qu'il manque de financement pour les projets | 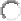 | 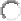 | 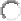 | 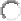 | 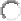 |
| Vous manquez de temps pour faire des études en plus de votre charge de travail actuelle | 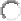 | 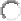 | 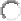 | 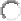 | 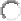 |
| Vous n'avez pas envie de faire de la recherche | 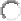 | 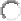 | 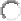 | 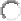 | 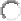 |
| On ne vous propose pas de faire des études | 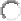 | 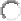 | 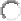 | 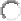 | 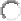 |
| Vous n'êtes pas au courant des différents projets existant | 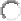 | 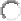 | 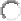 | 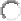 | 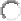 |
| Vous trouvez que ce n'est pas éthique de faire de la recherche sur des patients en soins palliatifs | 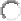 | 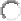 | 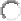 | 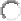 | 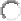 |
| Vous trouvez difficile de bien communiquer avec les patients pour avoir un consentement éclairé | 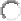 | 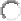 | 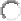 | 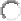 | 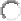 |
| Vous avez peur que les patients acceptent de participer car ils se sentent redevables | 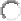 | 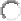 | 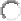 | 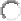 | 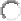 |
| Pour vous, l'état des patients peut parfois se dégrader trop vite pour faire de longues études | 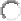 | 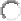 | 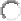 | 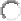 | 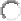 |
| Vous trouvez les études trop invasives (prises de sang supplémentaires, questionnaires, ...) |  |  |  |  |  |
| Vous avez l'impression de profiter du patient |  |  |  |  |  |

|  | Je suis en désaccord | Je suis un peu en désaccord | Neutre | Je suis un peu d'accord | Je suis d'accord |
| --- | --- | --- | --- | --- | --- |
| Vous avez l'impression que les résultats des études ne sont de toute façon pas appliqués en clinique |  |  |  |  |  |

1. Expérimentez vous une autre barrière à la recherche que celles susmentionnées?

.

Facilitateurs à la recherche

1. Quels sont les facilitateurs à la recherche

|  | Je suis en désaccord | Je suis un peu en désaccord | Neutre | Je suis un peu d'accord | Je suis d'accord |
| --- | --- | --- | --- | --- | --- |
| Vous êtes motivé.e à participer la recherche |  |  |  |  |  |
| Vous pensez que les patients pourront en bénéficier |  |  |  |  |  |
| Vous pensez que la recherche permet d'améliorer la qualité des soins |  |  |  |  |  |
| Vous pensez que les patients peuvent donner un sens à leur fin de vie à travers leur participation à des projets de recherche |  |  |  |  |  |
| Vous pensez que les patients sont autonomes (tant qu'ils ont leur capacité de discernement) et qu'il ne faut pas décider à leur place qu'ils ne peuvent pas participer à différents projets |  |  |  |  |  |
| Vous avez de l'expérience en recherche ou un réseau pour vous aider |  |  |  |  |  |
| Vous faites confiance aux comités d'éthique pour seulement accepter des projets de recherche corrects |  |  |  |  |  |

1. Expérimentez vous un autre facilitateur que ceux susmentionnés?

Pistes de solution

1. Qu'est ce qui vous aiderait pour participer à la recherche?

1. Si des pistes de solutions sont proposées dans votre service, seriez vous intéressé.e? Oui

Non

Je ne sais pas
